# The sylvian keyhole approach for surgical clipping of middle cerebral artery aneurysms: Technical nuance to the minipterional craniotomy

**DOI:** 10.3389/fsurg.2022.1078735

**Published:** 2022-12-20

**Authors:** Jonathan Rychen, Attill Saemann, Julian E. Gehweiler, Michel Roethlisberger, Jehuda Soleman, Gregor Hutter, Magdalena Müller-Gerbl, Luigi Mariani, Raphael Guzman

**Affiliations:** ^1^ Department of Neurosurgery, Basel University Hospital, University of Basel, Basel, Switzerland; ^2^ Translational Neurosurgical Anatomy Laboratory, Basel University Hospital, Basel, Switzerland; ^3^ 3D Print Lab, Department of Radiology, Basel University Hospital, University of Basel, Basel, Switzerland; ^4^ Faculty of Medicine, University of Basel, Basel, Switzerland; ^5^ Institute of Anatomy, University of Basel, Basel, Switzerland

**Keywords:** sylvian keyhole, minipterional, MCA aneurysm, clipping, cerebrovascular neurosurgery

## Abstract

**Background:**

The minipterional (MPT) craniotomy is a workhorse approach for clipping of middle cerebral artery (MCA) aneurysms. Because it aims to reach the skull base, traction on the temporal muscle is required. As a result, patients may suffer from transient postoperative temporal muscle discomfort. The sylvian keyhole approach (SKA) represents an alternative craniotomy for the clipping of MCA aneurysms. The aims of this study are to describe the operative technique of the SKA and to discuss the benefits and disadvantages compared to the MPT craniotomy.

**Methods:**

In this technical note, we report the experience gained with the SKA. This experience was acquired with virtual reality, 3D-printed models, and anatomical dissections. We also present two clinical cases.

**Results:**

The SKA is centered on the distal sylvian fissure and tailored toward the specific MCA aneurysm. Traction to the temporal muscle is not necessary because access to the skull base is not sought. With the SKA, dissection of the MCA is performed from distal to proximal, aiming for a proximal control at the level of the M1-segment. The limen insulae was identified as a key anatomical landmark for approach selection. The SKA offers good surgical maneuverability when the aneurysm is located at the level or distal to the limen. The MPT craniotomy, however, remains the most appropriate approach when the aneurysm is located proximal to the limen.

**Conclusion:**

The SKA represents a feasible and innovative alternative approach to the MPT craniotomy for surgical clipping of unruptured MCA aneurysms located at the level or distal to the limen insulae.

## Introduction

The classic pterional craniotomy, as described by Yasargil, has been the workhorse approach for surgical clipping of anterior circulation aneurysms in the last 40 years (1). In recent years, minimally invasive alternative approaches to the pterional craniotomy have become popular (2). The minipterional (MPT) craniotomy was primarily developed to improve cosmetic and functional outcomes by reducing temporal muscle atrophy, painful mastication, and scar length (3–5). Despite these technical improvements, a certain degree of transient postoperative painful mastication and temporomandibular joint dysfunction remained with the MPT approach (2, 6). In line with the pterional craniotomy, the MPT approach aims to give access to the skull base for proximal control of the internal carotid artery (ICA) and opening of the subarachnoid basal cisterns. Because the skin incision is located behind the temporal hair line, significant traction on the temporal muscle remains necessary to reach the skull base at the level of the lateral sphenoid ridge. This approach-related manipulation of the muscle may cause transient postoperative mastication discomfort. This frequently underestimated suboptimal patient outcome has led us to search for alternatives to the MPT craniotomy. The sylvian keyhole approach (SKA) represents an innovative tailored craniotomy for surgical clipping of middle cerebral artery (MCA) aneurysms (7). It is centered on the sylvian fissure and the MCA aneurysm, and does not aim to reach the skull base, defining it as a convexity craniotomy. Thus, less traction on the temporal muscle is needed and drilling of the lateral sphenoid ridge is not necessary. This technical note aims to describe the operative technique of the SKA and to discuss the potential benefits and disadvantages compared to the MPT craniotomy.

## Methods

This study is a technical note reporting the experience gained with the SKA. This experience was acquired with virtual reality (VR), 3D-printed models and anatomical dissection analyses. We also present two illustrative clinical cases. Extent of skin incision and bone flap, temporal muscle manipulation as well as surgical maneuverability to the MCA bifurcation and to the basal cisterns are described.

### Virtual reality

For VR analyses, we used the SpectroVR software developed at the Department of Biomedical Engineering, University of Basel. It is CE certified as a class I medical device (Diffuse Inc., Chicago, USA). The SpectroVR software renders a fully immersive 360° 3D VR model based on patient-specific DICOM data. DICOM images of CT-angiograms and/or digital subtraction angiographies of patients with different located and shaped MCA aneurysms were used. For visualization and manipulation of the 3D VR model, we used a commercial VR headset and controller (HTC-Vive, Valve HTC headset, Valve Corp., New Taipei, Taiwan). The accessibility to different MCA aneurysms *via* a MPT and a SKA was investigated.

### 3D-printed models

CT-angiograms of patients with different MCA aneurysms were analyzed. The DICOM files were processed in a medically certified image processing software (Mimics®, Materialise, Leuven, Belgium) to generate a 3D volumetric reconstruction model of the skull, the brain, and the vasculature. A virtual MPT craniotomy was performed; the 3D volumetric reconstruction was then manufactured with a high-resolution full-color 3D-printer (ProJet 660 Pro, VisiJet PXL material, 3D Systems, Inc., USA). The same procedure was performed for the SKA. Using these 3D-printed models, surgical accessibility to different MCA aneurysms was compared between the MPT and the SKA.

### Anatomical dissections

Anatomical dissections were performed at the Translational Neurosurgical Anatomy Laboratory of the University Hospital of Basel, Switzerland. We performed SKA and MPT craniotomies on fresh-frozen injected cadaver heads. The location and size of the skin incisions and bony openings were documented. Binocular microscopy (Wild Heerbrugg Leica M690, Leica Microsystems, Heerbrugg, Switzerland) was used for intradural dissections and to assess surgical maneuverability to the MCA bifurcation and to the basal cisterns. Anatomical dissections were rendered in form of artist's drawings to enhance visualization and comprehension of the presented concepts.

### Clinical cases

We present two illustrative clinical cases of MCA aneurysm clippings, treated *via* a MPT craniotomy and *via* a SKA, respectively.

### Ethics

The study was approved by the local ethics committee review board (EKNZ 2021-01862).

## Results

**Figure 1:**
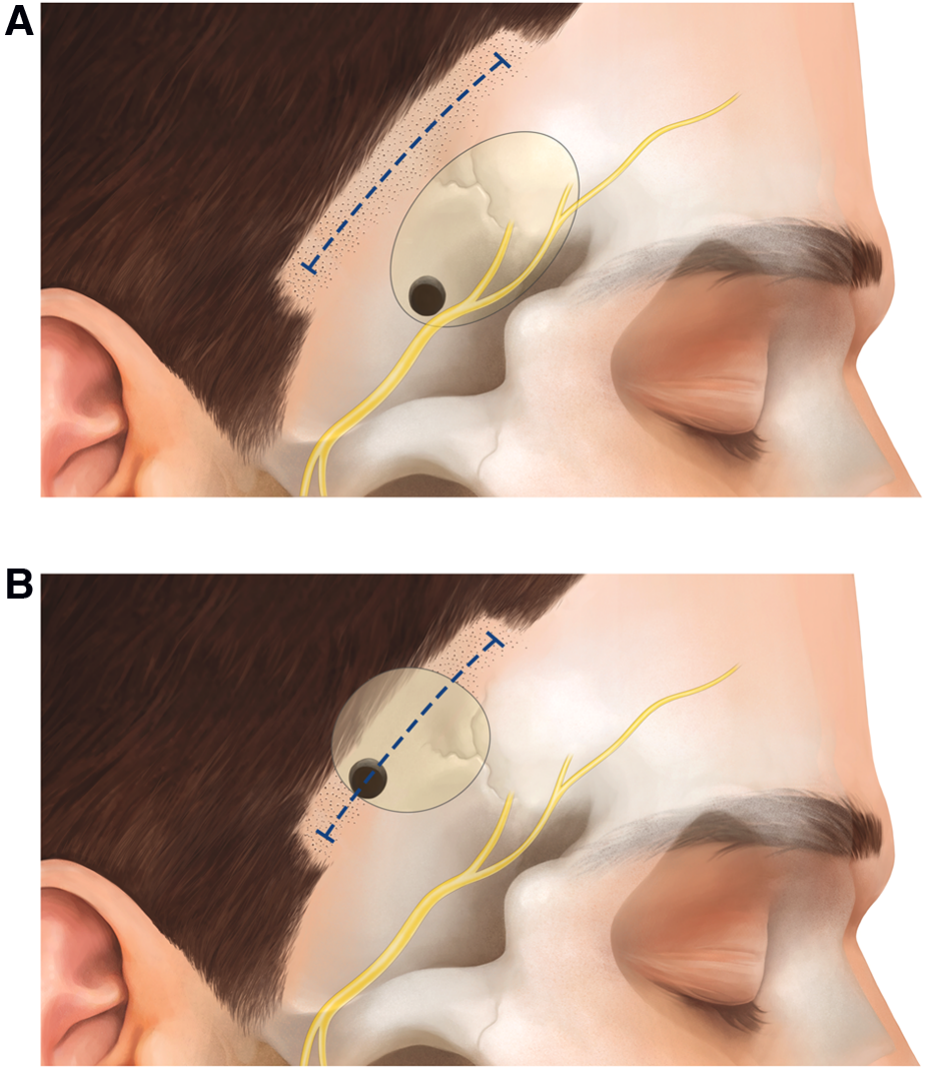
Schematic representation of the skin incision and craniotomy for the (**A**) MPT and the (**B**) SKA.

**Figure 2:**
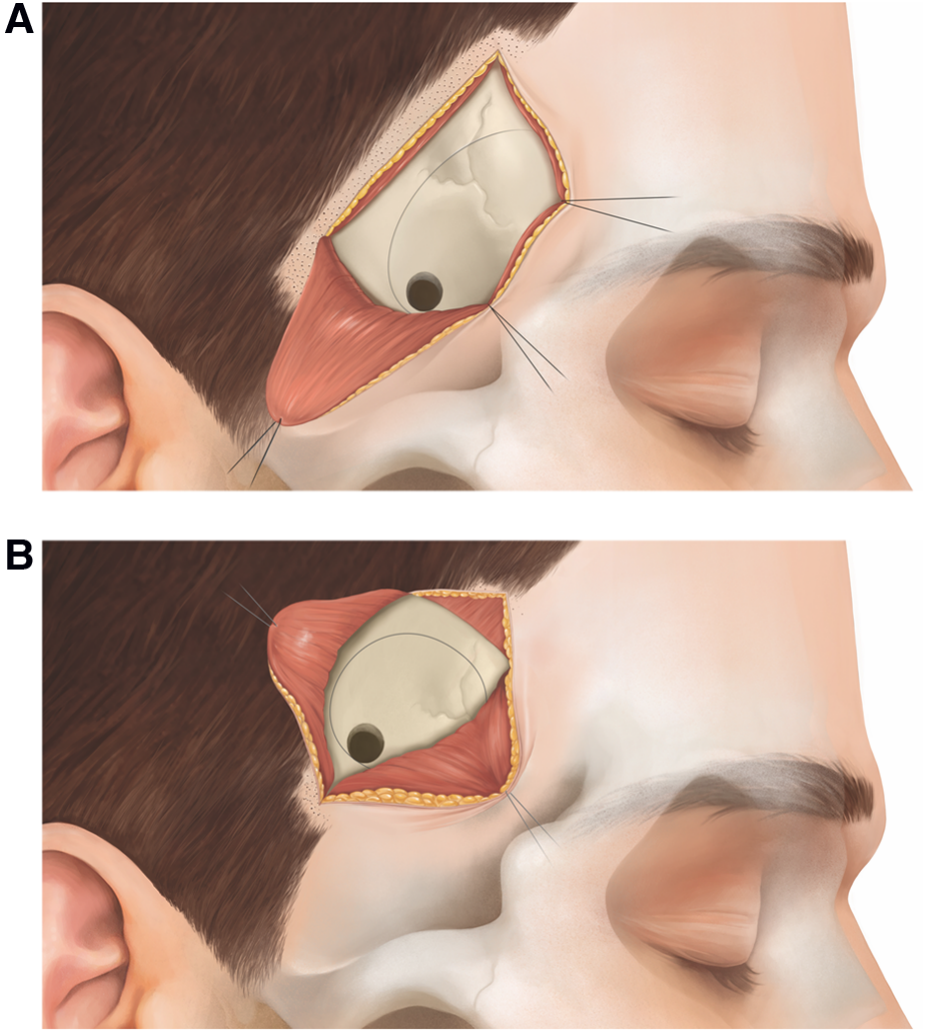
Schematic representation of the muscle incision/preparation for the (**A**) MPT and the (**B**) SKA.

**Figure 3:**
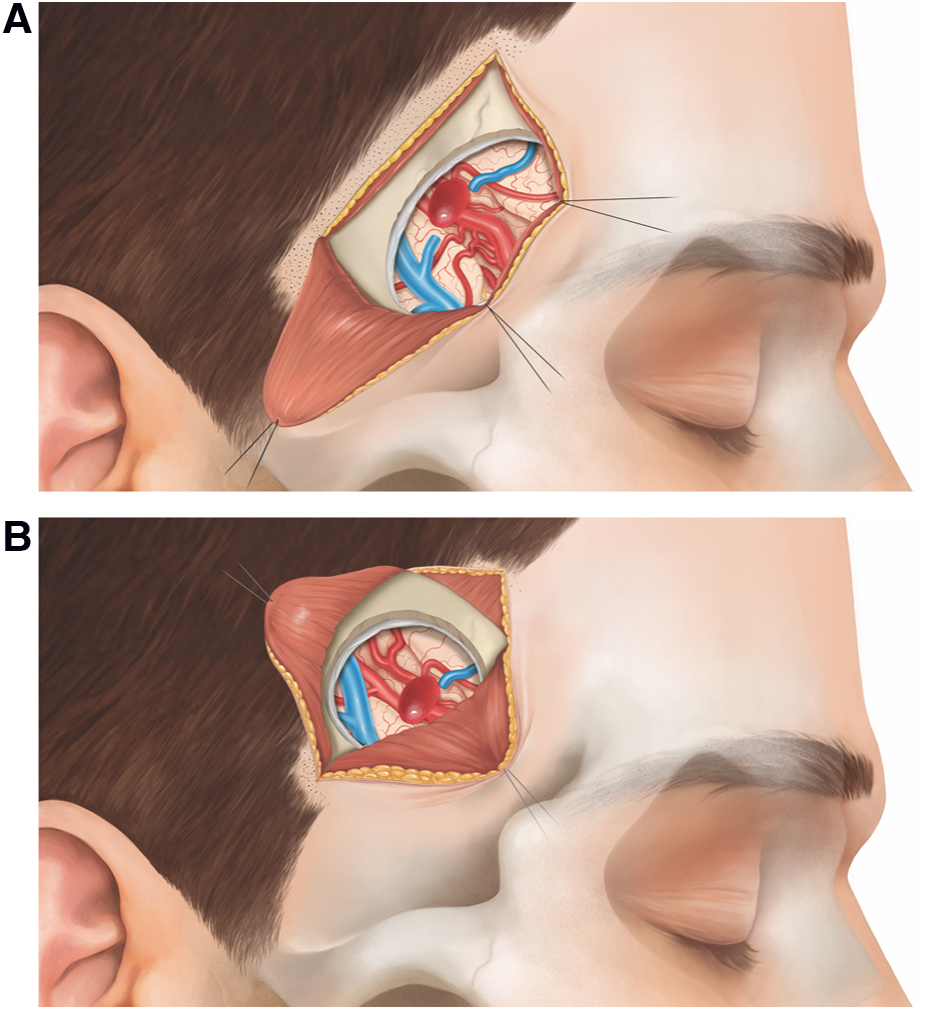
Schematic representation of a right-sided MCA bifurcation aneurysm, exposed *via* (**A**) MPT and (**B**) SKA.

**Figure 4:**
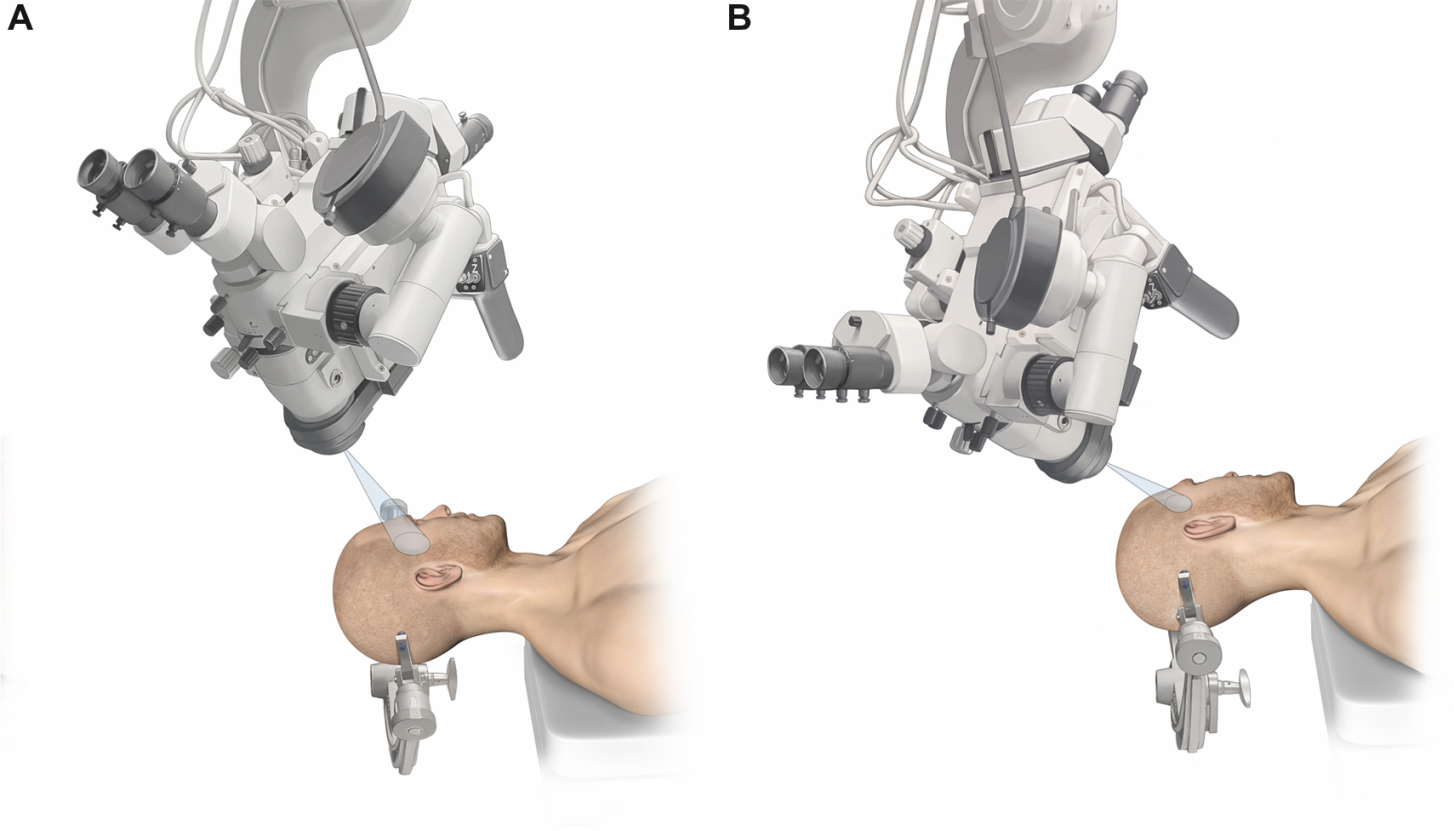
Operative microscope orientation and head rotation for the (**A**) MPT and the (**B**) SKA.

In the following, we describe the technical nuances of the MPT and the SKA (Figures 1–4). The presented concept applies to small, unruptured and non-complex MCA aneurysms.

### Surgical technique of the MPT craniotomy

The patient is placed in supine position with the head rotated 45° to the contralateral side (typical head rotation for MCA aneurysms). A 5.5 cm curvilinear incision is made behind the temporal hairline (Figure 1A). The scalp is elevated from the temporal fascia for a few centimeters until the fat pad is visualized. We prepare the temporal muscle using a subfascial dissection, although an interfascial dissection is also possible. The fascia is incised and elevated from the muscle. The temporal muscle is incised from the upper posterior corner of the muscle directly toward the sphenoid wing, and is retracted basally and posteriorly (Figure 2A). This detaches as much muscle as possible away from the lateral sphenoid wing. A 3 cm × 2.5 cm craniotomy is placed as anteriorly as possible to allow access to the skull base (Figure 2A). Subsequently, the lateral sphenoid wing is flattened by drill or kerrison punch until the meningo-orbital band is visualized. After durotomy, the proximal sylvian fissure is exposed (Figure 3A). Using the subfrontal corridor, the surgeon can easily open the carotid cistern, release CSF and gain proximal control at the level of the ICA. With the MPT craniotomy, proximal to distal as well as distal to proximal MCA dissection and aneurysm visualization are possible.

### Surgical technique of the SKA

The patient is positioned in supine position. Since the SKA is located more distally over the sylvian fissure, head rotation is more pronounced (about 60°) compared to the MPT craniotomy. For the SKA, the craniotomy is planned using the inline views of neuronavigation with a virtual tip extension of 5 cm on the navigation probe. Following an inside to outward concept, we first localize the distal M1 segment (where proximal control is sought), place the trajectory through the sylvian fissure and mark the entry point just behind the temporal hair line. Hence, the approach is centered on the sylvian fissure and tailored toward the specific MCA aneurysm. A short curvilinear incision of 4.5 cm is made behind the temporal hairline (Figure 1B). The temporal fascia is exposed and then incised together with the muscle (T-shape incision). The muscle is prepared in a submuscular (subperiosteal) fashion and gently reflected anteriorly and posteriorly (Figure 2B). A small myofascial cuff is left at the level of the superior temporal line for re-approximation at the end of the procedure. A small craniotomy of 2.5 cm × 2.5 cm is performed with a single burr hole placed at the inferior aspect of the exposed bone. Traction to the temporal muscle is not necessary because the craniotomy is placed just beneath the skin and muscle incision, and access to the skull base at the level of the sphenoid ridge is not sought (Figure 2B). The craniotomy is typically centered around the anterior squamous point. After durotomy, the sylvian fissure is exposed at the level of the anterior sylvian point, representing an ideal spot to open the sylvian fissure, due to the frequently observed enlargement of the sylvian cistern at this location. With the SKA, a distal to proximal dissection of the MCA is performed. The M2 branches of the MCA are followed toward the M1 segment, skipping carefully the aneurysm. Proximal control is gained at the level of the M1-segment, ideally from postero-medial in laterally pointing aneurysms and from lateral in anteriorly pointing aneurysms. After securing proximal control, the dissection is redirected toward the bifurcation to expose the aneurysm (Figure 3B).

### Approach selection for surgical clipping of MCA aneurysms

We identified the limen insulae as a key anatomical landmark for approach selection in the surgical clipping of MCA aneurysms. When the MCA aneurysm is located at the level or distal to the limen insulae, the SKA offers good surgical accessibility and maneuverability for safe aneurysm clipping because the aneurysm is located in the distal sylvian fissure. However, MCA bifurcation aneurysms located proximal to the limen insulae deep within the sphenoidal compartment of the sylvian fissure, are less accessible by a SKA. By orienting the microscope flatter toward the skull base when using the SKA (Figure 4), the surgeon is able to visualize and access the carotid cistern, dissect the fissure more deeply/proximally, and may treat aneurysms that are located proximally to the limen insulae. However, this is technically more challenging and depends on multiple factors such as brain relaxation, aneurysm size, and morphology. The MPT craniotomy offers good surgical accessibility and maneuverability for MCA aneurysms that are proximal as well as distal to the limen insulae. We found VR to be a valuable preoperative preparation tool to better understand the patient specific neurovascular architecture. As presented in the Supplementary Video, VR enables us also to evaluate the feasibility of the SKA for a particular aneurysm.

### Illustrative clinical cases

**Figure 5:**
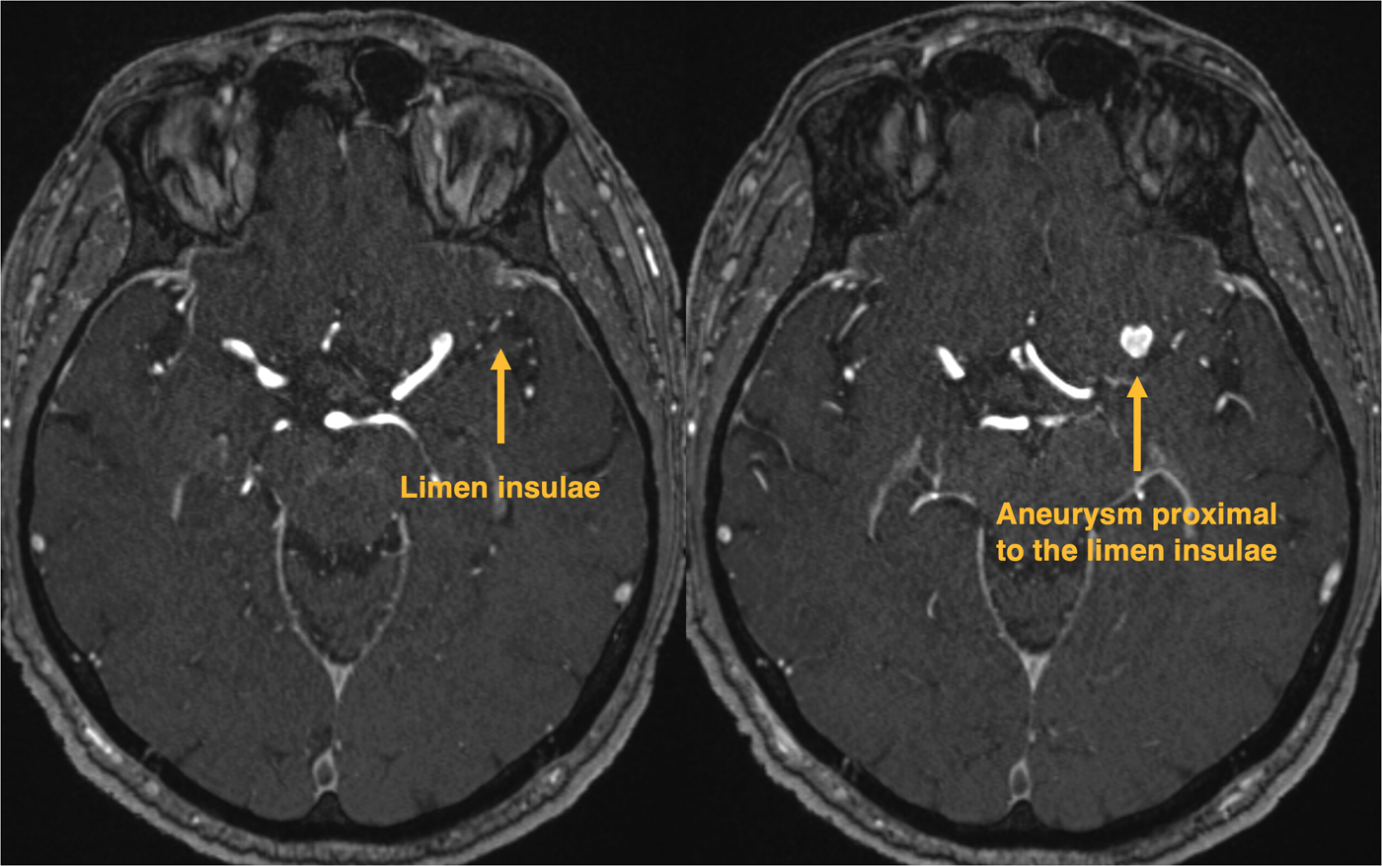
Minipterional case. Native time of flight angiography MRI with a left-sided MCA aneurysm, located proximally to the limen insulae.

Minipterional Case (Figure 5): 64-year-old man with an incidental unruptured 7 mm × 7 mm × 7 mm left-sided MCA bifurcation aneurysm. Due to this patient's relatively short M1 segment, the MCA bifurcation aneurysm is located proximally to the limen insulae and deep within the sphenoidal compartment of the proximal sylvian fissure (Figure 5). The MPT craniotomy was considered safer than a SKA to treat this particular aneurysm. Sylvian fissure dissection was performed from proximal to distal. Complete aneurysm clipping could be achieved without complications. However, postoperatively, the patient experienced a mild and transient temporal muscle discomfort for approximately 6 weeks.

**Figure 6:**
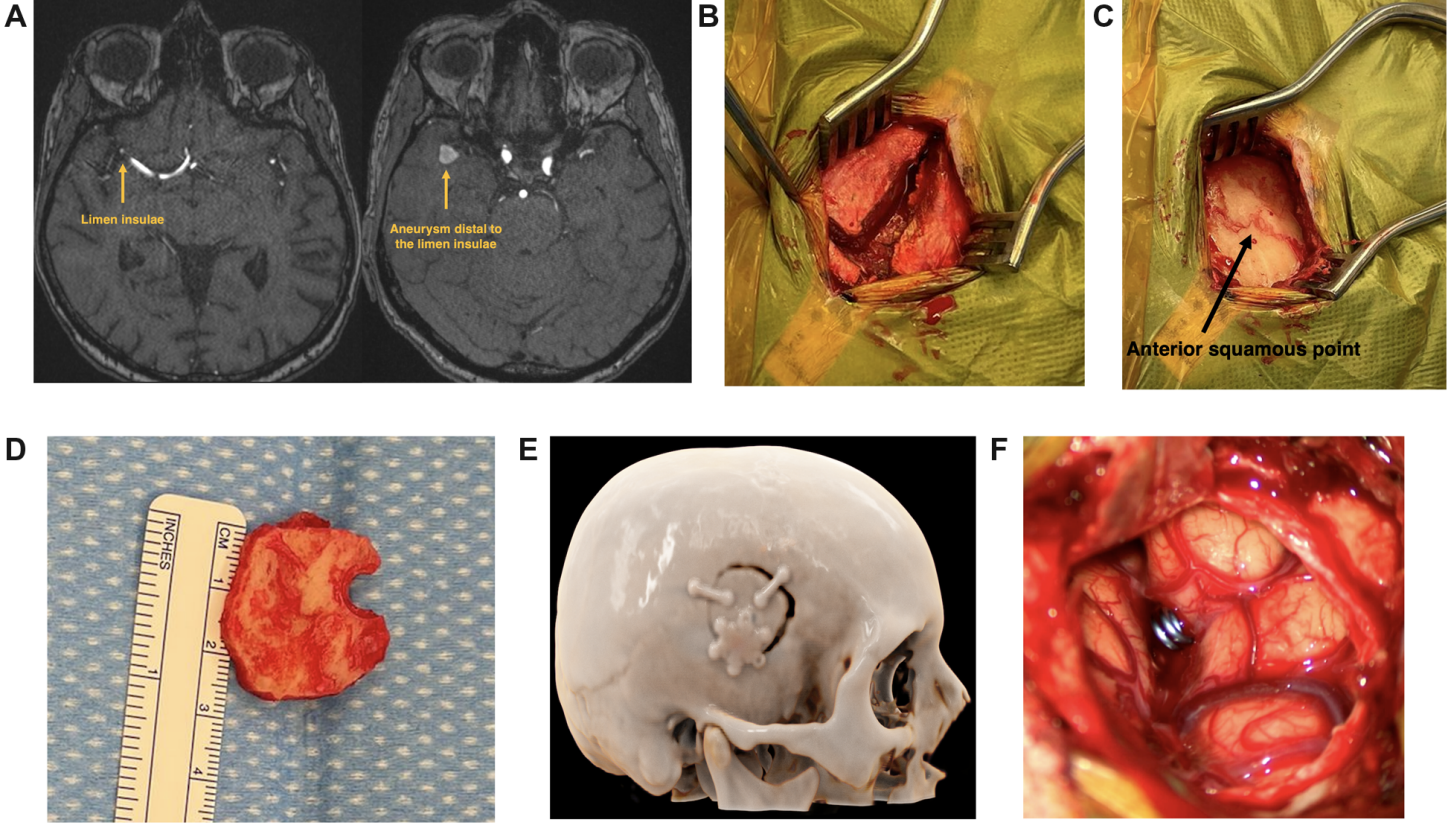
Sylvian keyhole approach case. (**A**) Native time of flight angiography MRI with a right-sided MCA aneurysm, located distally to the limen insulae. (**B**) Temporal muscle incision (T-shape incision). (**C**) The craniotomy is typically centered around the anterior squamous point. (**D**) 2.5 cm × 2.5 cm sized craniotomy. (**E**) Cinematic rendering of the postoperative CT-scan, showing location and size of the SKA. (**F**) Focal opening of the sylvian fissure (1.5 cm).

Sylvian Keyhole Case (Figure 6 and Supplementary Video): 79-year-old woman with an incidental unruptured 9 mm × 9 mm × 8 mm right-sided MCA bifurcation aneurysm. Due to this patient's relatively long M1 segment, the MCA bifurcation aneurysm is located distally to the limen insulae, making the SKA appropriate for surgical clipping of this aneurysm (Figure 6A). Surgical planning was performed with VR analysis of the patient's specific aneurysm and angioarchitecture (Supplementary Video). Using neuronavigation, the SKA was tailored toward the aneurysm. We performed a very focal opening of the sylvian fissure (Figure 6F) and did not aim to reach the carotid cistern. Proximal control was obtained at the level of the M1 segment. Complete aneurysm clipping could be achieved without complications. The patient did not experience any temporal muscle discomfort or temporomandibular dysfunction.

## Discussion

The MPT craniotomy was initially described by Nathal et al. (3) and popularized by Figueiredo et al. (4, 8). As the pterional craniotomy, the MPT approach aims to give access to the skull base. The lateral sphenoid ridge is drilled away to access the carotid cistern to release CSF and obtain proximal control of the ICA. Welling et al. (5) demonstrated a superior cosmetic outcome with the MPT approach compared to the pterional craniotomy due to reduced temporal muscle atrophy. However, a certain degree of postoperative temporal muscle discomfort and/or temporomandibular joint dysfunction remains clinically evident with the MPT approach (2, 6). This is mainly explainable with the significant traction and traumatization of the temporal muscle to reach the lateral sphenoid ridge.

Steiger et al. initially described the sylvian craniotomy for the treatment of MCA aneurysms in 2015; however, they performed this approach with a classical frontotemporal skin incision (7). We strived to develop a more tailored approach with less soft tissue trauma. We experienced the SKA as a valid alternative approach to the MPT craniotomy for clipping of MCA aneurysms located at the level or distal to the limen insulae. Indeed, the limen insulae represents a key anatomical landmark in approach selection. The SKA is typically located around the anterior squamous point (Figure 6C), which represents the bony landmark of the anterior sylvian point, lying in line with the limen insulae located more deeply within the sylvian fissure (9). The limen insulae separates the proximal sphenoidal from the distal insular portion of the sylvian fissure and underlies the genu of the MCA, where the vessel changes its direction to turn supero-posteriorly (10, 11). If the M1 segment is long, the MCA bifurcation aneurysm will be located distally to the limen insulae within the distal sylvian fissure (Figure 6A), making the SKA an optimal approach. With the SKA, drilling the lateral sphenoid ridge is not intended, making this approach potentially faster and safer. If the M1 segment is short, the MCA bifurcation aneurysm will be located proximally to the limen insulae in the depth of the sphenoidal sylvian compartment (Figure 5). In those cases, a MPT craniotomy is more appropriate, allowing a proximal to distal sylvian fissure dissection.

The presented concept highlights the effort to tailor minimally invasive approaches according to the anatomical location of the aneurysm, rather than to use a “one fits all” approach. With the same philosophy, Esposito et al. aimed to define a selection strategy for the treatment of MCA aneurysms (12, 13). They compared the MPT craniotomy with the lateral supraorbital approach. The approach selection is based on the depth of the aneurysm within the sylvian fissure, similar to the concept we present. The authors suggest using the lateral supraorbital approach when the aneurysm is <15 mm from the M1 origin and the MPT craniotomy when the aneurysm is ≥15 mm from the M1 origin. The SKA offers an additional tailored approach for MCA aneurysms and advances the field further toward personalized surgical approaches. When choosing the approach for surgical clipping of an MCA aneurysm, multiple factors are considered: rupture status, localization, size, and morphology (14). The presented concept is applied for unruptured, small, and non-complex MCA aneurysms.

The development of the SKA results from a modern process, beginning with VR and 3D-printed-models, going through anatomical dissections in the lab, and finally resulting in a clinical application. Well-designed cadaver-based morphometric anatomical analyzes are often demanding due to the needed setup. New technologies as VR broaden the spectrum and the possibilities of anatomical studies (15).

In our clinical practice, we use VR mainly as a preoperative preparation tool. It enables us to get a better understanding of the patient specific neurovascular architecture. It helps also to evaluate the feasibility of the SKA for a particular aneurysm. We found it very helpful to simulate the planned craniotomy, appreciate the angle of attack, turn around the aneurysm and get an impression of the intraoperative view (Supplementary Video). This is particular helpful for younger neurosurgeons when preparing for surgery. So far, we didn't experience any disadvantages using VR, as it takes only about 5 min to prepare the standardized setup.

### Limitations

This technical note is a proof of concept. It is mainly limited by the absence of a detailed morphometric analysis, as it would be necessary for a comparative analysis of two surgical approaches. Furthermore, this study is limited by the lack of a large case series demonstrating the safety of the described approach for clipping of MCA aneurysms. Finally, the hypothesized reduction in temporal muscle trauma and discomfort with the SKA should be analyzed with a well-designed comparative clinical study.

## Conclusion

The SKA represents a feasible and innovative tailored alternative approach to the MPT craniotomy for surgical clipping of small and unruptured MCA aneurysms located at the level or distal to the limen insulae. Clinical studies are necessary to analyze intra- and postoperative parameters to define the potential benefits of this approach compared to the MPT craniotomy.

## Data Availability

The original contributions presented in the study are included in the article/**Supplementary Material**, further inquiries can be directed to the corresponding author.
